# Supplementary material for: Synergistic Effect between SARS-CoV-2 Wave and COVID-19 Vaccination on the Occurrence of Mild Symptoms in Healthcare Workers
Source: Vaccines (Basel). 2023 Apr 22;11(5):882. doi: 10.3390/vaccines11050882 (PMC10221995; doi:10.3390/vaccines11050882)

Supplementary Table S1. Structure of the database

Cognome				
Nome				
Sesso				
Data di Nascita				
Ruolo				
Reparto				
Policlinico				
Vax. Anti-Covid				
Data I Dose				
Data II Dose				
Data III Dose				
Data Positività				
Data Negatività				
Contagio 1-2-3				
Sintomatologia 0-1				
Tosse				
Febbre				
Faringite				
Rinite				
Sindrome Influenzale				
Cefalea				
Anosmia				
Ageusia				
Astenia				
Congiuntivite				
Artralgia/Artrite				
Mialgia				
Diarrea				

Dolori Addominali				
Nausea				
Vomito				
Dispnea				
Polmonite				
Linfoadenopatia				
Esantema				
Paralisi Flaccida				
Rigor Nucalis				
Ipertransaminasemia				
Ittero				
Emoraggie Cutanee				
Sepsi/Shok Settico				
Altro				
ricovero				

Supplementary Table S2. Logistic regression - Symptomatology

OUTCOME: SYMPTOMATOLOGY		
VARIABLES	***P***	***ODDS RATIO – (95% CI)***
Gender	0.040	1.566 (1.190 – 2.060)
Age	0.497	0.997 (0.988 – 1.007)
Vaccination	0.998	0.912 (0.523 – 1.587)

Supplementary Table S3. Symptoms according to the number of vaccination doses

VARIABLES	Number of doses				P
	0	1	2	3	
Cough	189 (40)	55 (24.8)	26 (14.9)	186 (22.1)	< 0.001
Fever	227 (48.1)	63 (28.4)	44 (25.1)	139 (16.5)	< 0.001
Pharyngitis	81 (17.2)	32 (14.4)	39 (22.3)	247 (29.4)	< 0.001
Rhinitis	87 (18.4)	68 (30.6)	47 (26.9)	206 (24.5)	0.003
Flu syndrome	94 (19.9)	30 (13.5)	6 (3.4)	8 (1)	< 0.001
Headache	114 (24.2)	33 (14.9)	21 (12)	132 (15.7)	< 0.001
Anosmia	125 (26.5)	20 (9)	4 (2.3)	3 (0.4)	< 0.001
Ageusia	115 (24.4)	14 (6.3)	5 (2.9)	4 (0.5)	< 0.001
Asthenia	86 (18.3)	16 (7.2)	20 (11.4)	72 (8.6)	< 0.001
Conjunctivitis	18 (3.8)	1 (0.5)	2 (1.1)	4 (0.5)	< 0.001
Arthralgia/Arthritis	104 (22.1)	25 (11.3)	7 (4)	25 (3)	< 0.001
Myalgia	119 (25.3)	23 (10.4)	10 (5.7)	31 (3.7)	< 0.001
Diarrhea	36 (7.6)	3 (1.4)	4 (2.3)	10 (1.2)	< 0.001
Abdominal Pain	9 (1.9)	0 (0)	1 (0.6)	0 (0)	< 0.001
Nausea	15 (3.2)	0 (0)	2 (1.1)	8 (1)	0.002
Vomiting	4 (0.9)	1 (0.5)	1 (0.6)	4 (0.5)	0.846
Dyspnea	21 (4.5)	3 (1.4)	3 (1.7)	4 (0.5)	< 0.001
Pneumonia	3 (0.6)	0 (0)	0 (0.0)	0 (0.0)	0.048
Number of symptoms					
0	93 (19.7)	69 (31.1)	65 (37.1)	300 (35.7)	
1-3	208 (44.3)	130 (58.6)	97 (55.4)	495 (58.9)	< 0.001
≥ 4	169 (36)	23 (10.4)	13 (7.4)	46 (5.5)	

Supplementary Figure S1. Histogram related to the epidemiological trend of infection over time

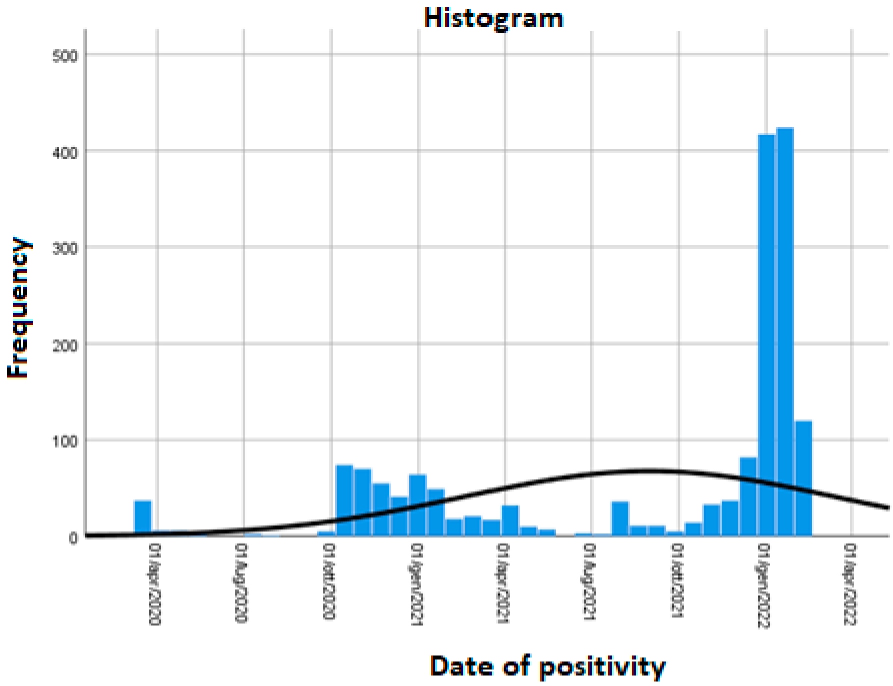

Supplement: Supplementary file 1 [file vaccines-11-00882-s001.zip › vaccines-2321155-supplementary.pdf]
